# Incidence of Venous Thromboembolism in Hospitalized Coronavirus Disease 2019 Patients: A Systematic Review and Meta-Analysis

**DOI:** 10.3389/fcvm.2020.00151

**Published:** 2020-08-06

**Authors:** Chi Zhang, Long Shen, Ke-Jia Le, Mang-Mang Pan, Ling-Cong Kong, Zhi-Chun Gu, Hang Xu, Zhen Zhang, Wei-Hong Ge, Hou-Wen Lin

**Affiliations:** ^1^Department of Pharmacy, Renji Hospital, School of Medicine, Shanghai Jiaotong University, Shanghai, China; ^2^Department of Cardiology, Renji Hospital, School of Medicine, Shanghai Jiaotong University, Shanghai, China; ^3^Department of Pharmacy, Nanjing Drum Tower Hospital, The Affiliated Hospital of Nanjing University Medical School, Nanjing, China; ^4^Department of Pharmacy, Roswell Park Comprehensive Cancer Center, Buffalo, NY, United States

**Keywords:** COVID-19, venous thromboembolism, pulmonary embolism, incidence, thromboprophylaxis, anticoagulation

## Abstract

**Background:** Emerging evidence shows that coronavirus disease 2019 (COVID-19) is commonly complicated by coagulopathy, and venous thromboembolism (VTE) is considered to be a potential cause of unexplained death. Information on the incidence of VTE in COVID-19 patients, however, remains unclear.

**Method:** English-language databases (PubMed, Embase, Cochrane), Chinese-language databases (CNKI, VIP, WANFANG), and preprint platforms were searched to identify studies with data of VTE occurrence in hospitalized COVID-19 patients. Pooled incidence and relative risks (RRs) of VTE were estimated by a random-effects model. Variations were examined based on clinical manifestations of VTE (pulmonary embolism-PE and deep vein thrombosis-DVT), disease severity (severe patients and non-severe patients), and rate of pharmacologic thromboprophylaxis (≥60 and <60%). Sensitivity analyses were conducted to strengthen the robustness of results. Meta-regression was performed to explore the risk factors associated with VTE in COVID-19 patients.

**Results:** A total of 17 studies involving 1,913 hospitalized COVID-19 patients were included. The pooled incidence of VTE was 25% (95% CI, 19–31%; *I*^2^, 95.7%), with a significant difference between the incidence of PE (19%; 95% CI, 13–25%; *I*^2^, 93.2%) and DVT (7%; 95% CI, 4–10%*; I*^2^, 88.3%; *P*_*interaction*_ < 0.001). Higher incidence was observed in severe COVID-19 patients (35%; 95 CI%, 25–44%; *I*^2^, 92.4%) than that in non-severe patients (6%; 95 CI%, 3–10%; *I*^2^, 62.2%; *P*_*interaction*_ < 0.001). The high rate of pharmacologic thromboprophylaxis in COVID-19 patients (≥60%) was associated with a lower incidence of VTE compared with the low pharmacologic thromboprophylaxis rate (<60%) (19 vs. 40%; *P*_*interaction*_ = 0.052). Severe patients had a 3.76-fold increased risk of VTE compared with non-severe patients (RR, 4.76; 95% CI, 2.66–8.50; *I*^2^, 47.0%). Sensitivity analyses confirmed the robustness of the primacy results.

**Conclusions:** This meta-analysis revealed that the estimated VTE incidence was 25% in hospitalized COVID-19 patients. Higher incidence of VTE was observed in COVID-19 patients with a severe condition or with a low rate of pharmacologic thromboprophylaxis. Assessment of VTE risk is strongly recommended in COVID-19 patients, and effective measures of thromboprophylaxis should be taken in a timely manner for patients with high risk of VTE.

## Introduction

Coronavirus disease 2019 (COVID-19) has spread globally, resulting in an unprecedented health crisis. As of 5 July 2020, there has been 11,125,245 cases of COVID-19 worldwide, of which 528,204 patients have died (1). Remarkably, emerging evidence shows that COVID-19 is commonly complicated by coagulopathy, and venous thromboembolism (VTE) is considered to be a potential cause of unexplained death, especially in severe COVID-19 patients (2, 3). A variety of potential risk factors of VTE exist among COVID-19 patients, including virus infection, respiratory failure, mechanical ventilation, and the use of a central venous catheter (4). The occurrence of VTE in COVID-19 patients, which includes pulmonary embolism (PE) and deep vein thrombosis (DVT), has been reported in several studies (5, 6). Thrombotic complications have been found in 31% of Intensive Care Unit (ICU) patients with COVID-19 in a Dutch teaching hospital (5), while 23% of PE incidence has been reported in a French hospital (7). At present, the incidence of VTE in this viral infection remains uncertain, however, understanding the precise incidence of VTE in COVID-19 patients is critically important for decision making on thromboprophylaxis. Accordingly, the present study summarizes all available evidence for a comprehensive and rigorous systematic review focused on VTE incidence in hospitalized COVID-19 patients, thus providing a panoramic view of this issue.

## Methods

This study was performed according to the standards of the Cochrane Handbook and the Preferred Reporting Items for Systematic Reviews and Meta-Analyses (PRISMA) statement (8). All supporting data is available within the article and the Supplementary File.

### Data Sources and Searches

The databases of PubMed, Embase, Cochrane Library databases, as well as the Chinese databases of the China National Knowledge Infrastructure (CNKI), China Science and Technology Journal Database (VIP), and the WANFANG databases were electronically searched from inception to 8 May 2020, using search terms related to COVID-19. Full details of the search terms used are presented in Table S1. Preprint articles were retrieved from MedRxiv (https://www.medrxiv.org), BioRxiv (https://www.biorxiv.org), and SSRN (https://www.ssrn.com). The references of identified records were also screened manually to find any further relevant articles.

### Study Selection and Outcomes

To be included, studies had to meet the following entry criteria: (1) included SARS-CoV-2 infected and hospitalized adult patients; (2) reported the data of VTE, PE, or DVT confirmed by computed tomography pulmonary angiography (CTPA) and/or ultrasonography. Two authors (CZ and LS) independently reviewed titles and abstracts of all studies, and assessed full texts of retrieved studies, with any discrepancies being resolved *via* consultation with a third author (ZG). The primary outcomes of this study were the incidence of VTE in hospitalized COVID-19 patients and corresponding relative risk in comparison between severe and non-severe patients. COVID-19 disease severity was defined according to the Clinical Management of COVID-19 (Interim guidance 27 May 2020) released by the World Health Organization (WHO). Criteria for severe cases included any of the following: (1) Respiratory rate >30 per min; (2) blood oxygen saturation (SPO2) <93% at rest; (3) partial pressure of arterial oxygen to fraction of inspired oxygen ratio <300; or (4) more than 50% of lung infiltrates within 24–48 h. Patients needing mechanical respiratory support or presenting with septic shock or multiple organ dysfunction or failure constituted critical cases.

### Data Extraction and Quality Assessment

All data from eligible studies were abstracted using *a priori* designed form, which included study characteristics (study name; country and period; population and number), clinical characteristics (mean age; gender ratio; previous VTE; the comorbidities of hypertension, diabetes, and cancer; and pharmacologic thromboprophylaxis rate), and data on VTE (occurrence number and total number of COVID-19 patients). The pharmacologic thromboprophylaxis rate was calculated as follows: the number of COVID-19 patients who received prophylactic anticoagulants (e.g., low molecular weight heparin [LMWH] or unfractionated heparin intravenously [UFH])/ total number of COVID-19 patients in the study. A rate of ≥60% was considered as a high proportion of pharmacologic thromboprophylaxis. The methodological quality of included studies was assessed according to the Newcastle-Ottawa Scale (NOS) (9). The NOS was modified according to our study design with a total of eight scores and the following six dimensions: (1) representative of the cases; (2) ascertainment of the exposure; (3) ascertainment of the outcome; (4) ascertainment of the outcome for quality control; (5) control for factors of age and gender; and (6) control for factors related to VTE. A study could receive a maximum of one point for the first four dimensions and a maximum of two points for the last two dimensions. Total scores with ≥ 5 points represented a relatively good quality.

### Data Synthesis and Statistical Analysis

All statistical analyses were performed using Stata version 13.0 (Statacorp, College Station, Texas, USA). The pooled incidence of VTE in hospitalized COVID-19 patients and associated 95% confidence intervals (95%CI) was calculated using a random-effects model, and relative risks (RRs) of VTE occurrence comparing severe with non-severe patients was also calculated. Heterogeneity among studies was assessed using the Cochran *Q*-test and *I*^2^ index, with *I*^2^ > 50% indicating considerable heterogeneity (10). Subgroup analysis was conducted by different manifestations of VTE (PE and DVT), severity of illness (severe patients and non-severe patients), and the pharmacologic thromboprophylaxis rate of patients (<60 and ≥60%). The interaction analysis (*P* for interaction) was applied to evaluate the risk difference of different subgroups. To strengthen the robustness of the results, leave-1-out sensitivity analyses were performed to explore whether a single study had an excessive influence on VTE incidence. Meta-regression was conducted to explore the potential risks associated with VTE. Funnel plots and Begg's test and Egger's test were carried out to qualitatively and quantitatively evaluate the presence of publication bias when more than 10 studies were available in a single analysis (11).

## Results

### Study Selection and Study Characteristics

**Figure 1:**
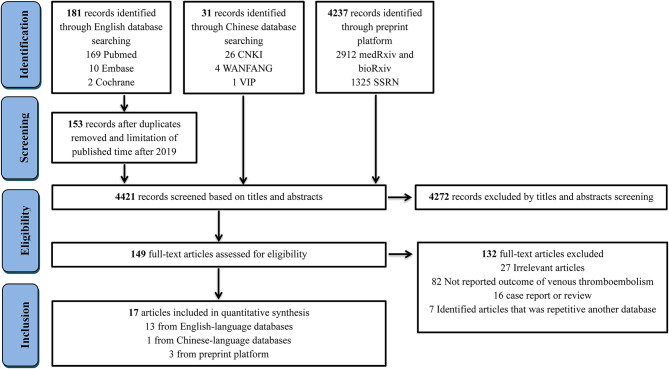
Flow diagram for the selection of eligible studies. CNKI, China National Knowledge Infrastructure; VIP, China Science and Technology Journal Database.

The process of study selection is outlined in Figure 1. A total of 4,449 records were identified through database searching, with 181 being from English-language databases, 31 from Chinese-language databases, and 4,237 from preprint platforms. Through reviewing the titles and abstracts, 28 duplicates were removed, and 4,272 records were excluded. The remaining 149 full-text articles were reviewed and 132 articles were excluded for the following reasons: irrelevant articles (*n* = 27), articles not reporting outcome of VTE (*n* = 82), case report or meta-analyses (*n* = 16), and repetition with another database (*n* = 7). Eventually, 17 retrospective studies (1, 5–8, 12–23) involving 1,913 hospitalized COVID-19 patients were included, 13 being from English-language databases, one from a Chinese-language database, and three from preprint platforms. Among them, six studies reported on patients in China, while 11 studies reported on patients in Europe, including the Netherlands, France, and Italy. The sample size of the involved studies varied from 16 to 420 patients. The detailed characteristics of included studies are presented in Table 1. Information of VTE and potential risk factors are summarized in Table S2. Of 17 studies, nine involved patients who had clinical suspicion of VTE and six included patients who were screened by CT or ultrasound. One study involved a population with both clinical suspicion and screening and one did not report the related information. Among 17 studies, 10 reported on patients who were prophylactically treated with anticoagulant therapy (Table S3), of which, five studies reported the dosage of LMWH (2,850 IU once to 6,000 IU twice). A high pharmacologic thromboprophylaxis rate was reported in seven studies, ranging from 66.7 to 100%.

**Table 1:** Characteristics of the included studies.

**Study**	**Country/period**	**Population/number**	**Mean age (*y*)**	**Male (%)**	**Previous VTE (%)**	**Hypertension (%)**	**Diabetes (%)**	**Cancer (%)**	**Pharmacologic thromboprophylaxis rate (%)**
Beun et al. (12)	Netherlands/NR	ICU/75	60.5	50	NR	NR	NR	NR	NR
Bi et al. (8)	China/2020.1.11–2020.3.10	Mild-moderate and severe-critical/420	NR	47.6	NR	11.7	5.7	0.2	NR
Chen et al. (1)	China/2020.1.1–2020.2.29	Mild-moderate and severe-critical/25	65	60	4	40	20	NR	80
Cui et al. (13)	China/2020.1.20–2020.3.22	ICU/81	59.9	46	NR	25	10	NR	0
Ding et al. (14)	China/2020.1.1–2020.2.3	NR/56	54.6	53.57	NR	NR	NR	NR	NR
Grillet et al. (7)	France/2020.3.15–2020.4.14	Non-ICU and ICU/100	66	70	NR	NR	20	20	NR
Helms et al. (15)	France/2020.3.3–2020.3.31	ICU/150	63	81.3	5.3	NR	NR	NR	66.7
Klok et al. (5)	Netherlands/NR-2020.4.5	ICU/184	64	76	NR	NR	NR	2.7	100
Li et al. (18)	China/2020.1.1–2020.2.13	Suspected PE/24	63	63.6	0	63.6	NR	NR	NR
Llitjos et al. (19)	France/2020.3.19–2020.4.11	ICU/26	68	77	4	85	NR	NR	31
Lodigiani et al. (20)	Italy/2020.2.13–2020.4.10	Non-ICU and ICU/362	66	68	0	47.2	22.7	6.4	Overall: 81.2%; ICU: 100%; Non-ICU: 78.3%
Leonard-Lorant et al. (17)	France/2020.3.1–2020.3.31	Non-ICU and ICU/106	63.3	66	NR	NR	NR	NR	39.6
Middeldorp et al. (21)	Netherlands/NR-2020.4.12	Non-ICU and ICU/198	61	66	5.6	NR	NR	3.5	100
Poissy et al. (6)	France/2020.2.27–2020.3.31	ICU/107	NR	NR	0.93	NR	NR	NR	NR
Ranucci et al. (22)	Italy/2020.3.8-NR	ICU/16	61	93.75	0	NR	NR	NR	100
Tavazzi et al. (16)	Italy/2020.2.21-NR	ICU/54	68	83	NR	NR	NR	NR	100
Xing et al. (23)	China/NR	Moderate and severe-critical/20	NR	60	NR	NR	NR	NR	NR

*NR, not reported; ICU, intensive care unit; VTE, venous thromboembolism; PE, pulmonary embolism*.

### Study Quality

All included studies satisfied the following risk bias items: representative of the cases; ascertainment of the exposure; ascertainment of the outcome; and ascertainment of the outcome for quality control. In total, 13 studies (82.3%) presented both age and gender ratio of the included patients, while seven studies (41.2%) reported more than three clinical characteristics (two points) and eight studies (47.1%) reported one or two clinical characteristics (one point). Accordingly, all 17 included studies were considered as being of relatively good quality (Table S4).

### Incidence of VTE

**Figure 2:**
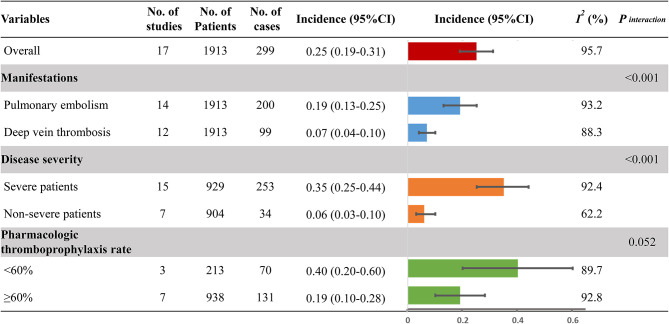
Incidence of venous thromboembolism. No., number; 95% CI, 95% confidence interval.

Figure 2 provides the full view of VTE incidence in hospitalized COVID-19 patients. The overall pooled incidence of VTE was 25% (95% CI, 19–31%; *I*^2^, 95.7%) (Figure S1). The incidence of PE and DVT was significantly different (*P*_*interaction*_ < 0.001), with the event rate being 19% (95% CI, 13–25%; *I*^2^, 93.2%; Figure S2) and 7% (95% CI, 4–10%; *I*^2^, 88.3%; Figure S3), respectively. Considering the disease severity of COVID-19, a higher incidence was observed in severe patients (35%; 95 CI%, 25–44%; *I*^2^, 92.4%; Figure S4) than that in non-severe patients (6%; 95 CI%, 3–10%; *I*^2^, 62.2%; Figure S5; *P*_*interaction*_ < 0.001). Because anticoagulation for thromboprophylaxis could decrease the occurrence of VTE, the high pharmacologic thromboprophylaxis rate of above 60% was associated with a lower incidence of VTE (19%; 95 CI%, 10–28%; *I*^2^, 92.8%; Figure S6) when compared to the low pharmacologic thromboprophylaxis rate of below 60% (40%; 95 CI%, 20–60%; *I*^2^, 89.7%; Figure S6; *P*_*interaction*_ = 0.052). Sensitivity analysis, by removing a single study at a time, confirmed the robustness of primacy results (Table S5).

### Comparison of VTE Risk With Severe vs. Non-severe Patients

**Figure 3:**
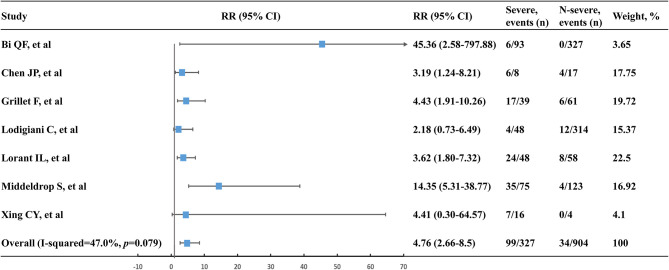
Venous thromboembolism risk of severe patients vs. non-severe patients. n, number; RR, relative risk; 95% CI, 95% confidence interval.

A total of 99 VTE events were found in 327 severe patients with the event rate of 30.3%. Comparatively, 34 VTE events were observed in 904 non-severe patients with a low event rate of 3.8%. Accordingly, severe patients were at a higher risk of VTE compared to non-severe patients (RR, 4.76; 95% CI, 2.66–8.50; *I*^2^, 47.0%) (Figure 3). Leave-1-out sensitivity analysis was consistent with the primacy result (Table S6).

### Risk Factors Associated With VTE

Meta-regression was conducted to assess the potential risk factors associated with VTE incidence. Seven variables (mean age, gender ratio, previous VTE, the comorbidities of hypertension, diabetes, cancer, and pharmacologic thromboprophylaxis rate) were evaluated, and none of them were detected to be related to the incidence of VTE (Table S7).

### Publication Bias

The funnel plots for VTE incidence as well as PE and DVT incidence were all asymmetrical on visual inspection. The corresponding *P*-values for the Egger's test were <0.001, <0.001, and <0.001, and the corresponding *P*-values for the Begg's test were 0.26, 0.009, and 0.003, respectively (Figure S7). Because of limited study numbers in comparison to severe and non-severe patients (seven studies), a funnel plot was not performed.

## Discussion

The true incidence of VTE in patients with COVID-19 remains uncertain. This is the first systematic review and meta-analysis to provide a comprehensive overview of VTE occurrence based on 17 retrospective studies involving 1,913 hospitalized COVID-19 patients. The overall VTE incidence was 25%, with the event rate of PE and DVT being 19 and 7%, respectively. Considering disease severity, a higher incidence was observed in severe patients (35%) than in non-severe patients (6%). Moreover, a high pharmacologic thromboprophylaxis rate of above 60% was associated with a lower incidence of VTE (19%) compared to a low pharmacologic thromboprophylaxis rate of below 60% (40%). Severe patients had a 3.76-fold increased risk of VTE compared to non-severe patients. The prevalence of VTE in COVID-19 patients seemed to be high, especially for severe patients. Therefore, it is important to improve the awareness of thromboprophylaxis for COVID-19 infection.

It was reported that a high proportion of severe and critically ill COVID-19 patients showed major coagulation disorders (24, 25). In our meta-analysis, a higher incidence of VTE was also found in severe patients (35%) than in non-severe patients (6%), with a risk ratio of 4.76. The results were similar to recent preliminary studies on COVID-19, in which the event rate of VTE for ICU patients was 2.18–4.42 folds than that of general ward patients (7, 20). In fact, the prevalence of VTE appeared to be higher in ICU patients than in patients in other disease conditions, with the mean rate of VTE diagnosis being 12.7% (26). The higher risk of VTE in ICU patients mainly resulted from both individual patient related risk factors (e.g., age, history of VTE, cancer) and ICU-specific risk factors (e.g., sedation, immobilization, central venous catheters) (27), therefore, pharmacological VTE prophylaxis is strongly recommended to critically ill patients by clinical guidelines (28). It is speculated that COVID-19 is probably an additional risk factor for VTE in hospitalized patients (29). As for severe or critically ill patients with COVID-19, the release of large amounts of inflammatory mediators and the application of hormones and immunoglobulin might exacerbate the blood hypercoagulability (23, 30). Rapid deterioration in oxygen saturation and increased dead space ventilation could also be factors of VTE events (31). Moreover, severe COVID-19 patients could present with a high fever, dehydration, as well as immobilization (32), which might also lead to VTE (33, 34). Therefore, underestimated prevalence of VTE and inadequate thromboprophylaxis might exist among critically ill COVID-19 patients (35). It was reported that even on standard doses of thromboprophylaxis, the incidence of thrombotic complications was still as high as 31% for ICU patients with COVID-19 infection (5). Accordingly, routinely screening for VTE by CTPA or ultrasound, as well as the use of full-dose anticoagulation, are now recommended for critically ill COVID-19 patients by some experts.

To increase the awareness of thrombotic complications, the assessment of VTE risk should be strongly recommended, for the sake of taking timely and effective preventive measures for patients at high risk of VTE. It is recognized that prevention of VTE is required in all severe or critically ill patients in absence of anticoagulation contraindication (30, 36). For mild or moderate patients with COVID-19, determination of VTE risk might be exerted using the PADUA risk assessment model for medical patients and the CAPRINI prediction score for surgical patients, as there are currently no new VTE risk assessment models that are specialized for COVID-19 patients (30). Therefore, measures of thromboprophylaxis could be taken without delay in patients with high or moderate risk of VTE. Importantly, dynamic and repeated assessment for thrombotic risk should also be conducted in the course of treatment, including routine coagulation tests, concomitant medications, and invasive procedures, to adjust the antithrombotic regimen in a timely manner. Furthermore, the regular evaluation of bleeding risk should not be neglected in COVID-19 patients, and should be carefully balanced against the risk of thrombosis.

Anticoagulants are definitely the cornerstone for VTE prevention. Therefore, COVID-19 patients with high VTE risk should receive pharmacologic thromboprophylaxis, unless there are absolute contraindications (37, 38). As found in this study, the high pharmacologic thromboprophylaxis rate of above 60% was associated with a lower incidence of VTE (19 vs. 40%) compared with the low pharmacologic thromboprophylaxis rate of below 60%. A recent study involving 449 severe COVID-19 patients revealed that LMWH users appeared to be associated with better prognosis compared with non-users (39). Remarkably, prophylactic daily LMWH or twice daily subcutaneous UFH are now recommended for all hospitalized COVID-19 patients by the WHO as well as the International Society on Thrombosis and Haemostasis (ISTH) (23, 37, 40). Nevertheless, prophylactic dose of anticoagulation is supposed to be insufficient to contrast the hypercoagulable state presented by many COVID-19 patients in response to a cytokine storm syndrome (41). A substantial number of patients with standard doses of thromboprophylaxis could still suffer from thrombotic complications, which was also observed in some studies involved in our meta-analysis (5, 16, 20). These findings are strongly suggestive of a higher dose of anticoagulation for patients at high risk of VTE. Given the relatively high VTE occurrence found in early reports, it might therefore be appropriate to conduct a universal thromboprophylaxis strategy for all hospitalized COVID-19 patients (42), however, more evidence is needed to support these considerations.

## Strengths and Limitations

This is the first systematic review and meta-analysis that estimates the relatively precise incidence of VTE in hospitalized COVID-19 patients. A comprehensive search of English-language databases, Chinese-language databases, and preprint platforms was conducted, and a revised NOS tool was used to assess the study quality appropriately. Subgroup analyses were conducted by clinical manifestations, disease severity, as well as pharmacologic thromboprophylaxis rate, to explore the differences on VTE incidence. Nevertheless, several intrinsic limitations still remained in this study. First, in this meta-analysis, 17 retrospective studies with three being from preprint platforms were included. Because of the unexpected outbreak of COVID-19, timely information and initial experiences are urgently needed by medical workers to decide on the most optimal therapy for infected patients. Given that journal publications requires peer review and is a time-consuming process, preprints might provide a mechanism for rapidly communicating research, although they are recognized as being less reliable than peer reviewed journal publications. In order to perform a comprehensive meta-analysis, we analyzed as many studies as we could find in this field. Additionally, all studies included were retrospective, which could inevitably introduce heterogeneity to the results. Further studies published in journals as well as high quality studies are therefore needed to obtain more reliable results. Second, given the difficulty of performing CTPA or ultrasonography under strict isolation, it might be difficult to fully illuminate the exact prevalence and nature of VTE in COVID-19. Third, patient-level information about comorbidities and concomitant medication was unavailable to explore the potential risk factors of VTE. Furthermore, whether patients were on thromboprophylaxis or not, as well as different pharmacologic thromboprophylaxis rates, could also contribute to heterogeneity. In addition, the association between the occurrence of VTE and coagulation indicators, such as D-dimers and fibrin degradation products, was not assessed in this study.

## Conclusion

This meta-analysis revealed that the estimated VTE incidence was 25% in hospitalized COVID-19 patients, with the incidence of PE and DVT being 19 and 7%, respectively. Higher incidence was observed in severe patients (35%) than in non-severe patients (6%). The high pharmacologic thromboprophylaxis rate was associated with a lower incidence of VTE compared with the low pharmacologic thromboprophylaxis rate. Assessment of VTE risk is therefore strongly recommended in COVID-19 patients, and effective measures of thromboprophylaxis should be taken for patients at high risk of VTE in a timely manner.

## Data Availability Statement

The original contributions presented in the study are included in the article/Supplementary Material, further inquiries can be directed to the corresponding author/s.

## Author Contributions

Z-CG and HX are the guarantors of the entire manuscript. CZ, LS, and K-JL contributed to the study conception and design, critical revision of the manuscript for important intellectual content, and final approval of the version to be published. M-MP, L-CK, ZZ, W-HG, and H-WL contributed to the data acquisition, analysis, and interpretation. All authors contributed to the article and approved the submitted version.

## Conflict of Interest

The authors declare that the research was conducted in the absence of any commercial or financial relationships that could be construed as a potential conflict of interest.
