# Supplementary material for: Incidence of Venous Thromboembolism in Hospitalized Coronavirus Disease 2019 Patients: A Systematic Review and Meta-Analysis
Source: Front Cardiovasc Med. 2020 Aug 6;7:151. doi: 10.3389/fcvm.2020.00151 (PMC7423832; doi:10.3389/fcvm.2020.00151)
Supplement: Supplementary file 1 [file Data_Sheet_1.PDF]

SUPPLEMENTAL FILE

Title: Incidence of venous thromboembolism in hospitalized Coronavirus Disease 2019 patients:

a systematic review and meta-analysis

Indexes

Table S1. Search strategy used in May 8, 2020 2

Table S2. Detailed information of VTE and associated potential risk factors in the included studies 4

Table S3. Pharmacologic thromboprophylaxis information of the included studies 7

Table S4. Quality scores of the included studies 11

Table S5. Leave-1-out sensitivity analysis for VTE incidence 13

Table S6. Leave-1-out sensitivity analysis for relative risk of severe patients vs. non-severe patients 14

Table S7. Univariable meta-regression for VTE incidence 15

Figure S1. Incidence of venous thromboembolism 17

Figure S2. Incidence of pulmonary embolism 18

Figure S3. Incidence of deep vein thrombosis 19

Figure S4. Incidence of venous thromboembolism in severe patients 20

Figure S5. Incidence of venous thromboembolism in non-severe patients. 21

Figure S6. Incidence of venous thromboembolism by anticoagulation rate 22

Figure S7. Publication bias on the incidence of venous thromboembolism (A. venous thromboembolism; B. pulmonary embolism; C. deep venous thrombosis) 23

Table S1. Search strategy used in May 8, 2020

Literature databases	Search items	Items found
Pubmed	#1<br>COVID-19[Title/Abstract] OR 2019-nCov[Title/Abstract] OR novel coronavirus[Title/Abstract] OR Wuhan coronavirus[Title/Abstract] OR Wuhan pneumonia [Title/Abstract] OR SARS-CoV-2[Title/Abstract] OR coronavirus 2019[Title/Abstract]<br>#2<br>deep venous thrombosis [All Fields] OR deep venous thromboembolism [All Fields] OR pulmonary embolism [All Fields] OR venous thromboembolism [All Fields] OR thrombus [All Fields] OR thrombosis [All Fields] OR VTE [All Fields] OR PE [All Fields] OR DVT [All Fields] OR thrombotic [All Fields] OR thromboembolic [All Fields]<br>#1 AND #2	169
Embase	#1<br>'covid 19':ab,ti OR '2019 ncov':ab,ti OR 'novel coronavirus':ab,ti OR 'wuhan coronavirus':ab,ti OR 'wuhan pneumonia':ab,ti OR 'sars cov 2':ab,ti OR 'coronavirus 2019':ab,ti<br>#2<br>'deep venous thrombosis': all fields OR 'deep venous thromboembolism': all fields OR 'pulmonary embolism': all fields OR 'venous thromboembolism': all fields OR 'thrombus': all fields OR 'thrombosis': all fields OR 'VTE': all fields OR 'PE': all fields OR 'DVT': all fields OR 'thrombotic': all fields OR 'thromboembolic': all fields	10

	#1 AND #2 AND NOT ([embase]/lim AND [medline]/lim)	
Cochrane	#1<br>(COVID-19):ti,ab,kw OR (novel coronavirus):ti,ab,kw OR (Wuhan coronavirus):ti,ab,kw OR (Wuhan pneumonia):ti,ab,kw OR (SARS-CoV-2):ti,ab,kw OR (coronavirus 2019):ti,ab,kw<br>#2<br>(deep venous thrombosis): all text OR (deep venous thromboembolism): all text OR (pulmonary embolism): all text OR (venous thromboembolism): all text OR (thrombus): all text OR (thrombosis): all text OR (VTE): all text OR (PE): all text OR (DVT): all text OR (thrombotic): all text OR (thromboembolic): all text<br>#1 AND #2	2
Overall		181
Duplication		153

Table S2. Detailed information of VTE and associated potential risk factors in the included studies

**Study**	**Detailed VTE Information**	**Potential Risk factors**
Beun R, et al	35 patients were clinical suspicion of thrombo-embolic event and performed CT or ultrasound. 16 patients developed PE in segmental arteries and 4 in central artery. 3 patients had DVT.	ICU patients: 100%
Bi QF, et al	420 patients were screened by CT and ultrasound. 6 patients developed DVT.	ICU patients: 4.5%; age >70: 4.5%; coronary heart disease: 2.6%; cerebrovascular disease: 0.5%
Chen JP, et al	25 patients were suspected PE and 10 patients were PE after performing CTPA.	The median time from onset of COVID-19 symptoms to CTPA examination was 10 days. Cardiovascular disease: 16%; surgery 24%; previous DVT: 4%; cancer: 0%
Cui SP, et al	81 patients were screened by lower limb venous doppler ultrasound. 20 patients developed DVT, of which 8 patients died.	ICU patients: 100%; age⩾70: 22%; coronary heart disease: 12%
Ding Y, et al	81 patients were screened by CT scan. One patient was PE.	Not reported
Grillet F, et al	100 patients were screened by CT scan. 23 patients had acute PE.	Longer delay from symptom onset to CT diagnosis of pulmonary embolus (12 ± 6 versus 8 ± 5 days, p<0.001). requirement for mechanical ventilation (OR =3.8 IC95% [1.02 - 15], p=0.049) associated with acute pulmonary embolus. ICU patients: 39%; cardiovascular disease 39%; Malignancy 20%

Helms J, et al	99 Patients with suspected pulmonary embolism, based on their clinical had a CTPA done, either at the admission in ICU or during their stay. 25 patients showed PE.	Pulmonary embolism was diagnosed in median 5.5 days after ICU admission. ICU patients: 100%; cardiovascular diseases 48%; malignancies: 6%
Klok FA, et al	Diagnostic tests were only applied if thrombotic complications were clinically suspected. Among 184 patients, 25 patients had PE and 3 had DVT.	ICU patients: 100%; active cancer: 2.7%
Li T, et al	24 patients with elevated D-dimer and suspected to have PE were evaluated with CTA. 11 patients had PE.	Not reported.
Llitjos JF, et al	PE was systematically searched in patients with persistent hypoxemia or secondary deterioration. 26 consecutive patients with severe COVID-19 were screened for VTE. 18 patients had DVT and PE was diagnosed in 6 patients.	Days between symptom onset and admission were 7 days. ICU patients: 100%; active cancer: 0%; previous VTE: 4%; surgery less than 3 months: 4%
Lodigiani C, et al	VTE imaging test were performed in subjects with signs or symptoms of DVT or with an unexplained clinical worsening of the respiratory function. Two-point compression ultrasonography (CUS) was used on the ICU; whole-leg ultrasound was performed in symptomatic patients on the general ward. Among 362 patients, 10 had PE and 6 had DVT.	The median length of stay in the ICU was 12 days, the VTE event was diagnosed within 24 h of hospital admission. ICU patients: 100%; BMI⩾30: 24.1%; active cancer: 6.4%; coronary artery disease 13.9%; prior stroke: 5.2%; prior VTE: 3.1%; hormonal treatment: 1.0%; Aspirin: 24.5%: Vitamin K antagonists: 4.1%; Direct oral anticoagulants: 4.4%

Lorant IL, et al	The reason for CT angiography in these patients was suspicion of PE. Among 106 patients, 32 had PE.	ICU patients: 45.3%
Middeldorp S, et al	Screening for lower extremity DVT was performed in 55 patients (28%) during hospital stay, while CT pulmonary angiography for PE was only performed on indication (e.g. sudden worsening hypoxemia). VTE was symptomatic in 25 patients and detected incidentally or by screening in 14.	VTE was diagnosed after a median of 7 days after admission and symptomatic VTE also after a median of 7 days. Risk factors associated with VTE in univariable regression analyses were a higher white blood cell count, higher neutrophil-to-lymphocyte ratio and a higher D-dimer level. ICU patients: 37.9%; body weight more than 100kg: 14%; history of VTE: 5.6%; active cancer: 3.5%
Poissy J, et al	The criteria for decision to perform CTPA were suspicion of PE upon admission and/or acute degradation of hemodynamic or respiratory status. Of 107 patients, 22 were PE and 5 had DVT.	ICU patients: 100%
Ranucci M, et al	The patient population comprised 16 patients with a diagnosis of COVID-19 associated pneumonia and ARDS, admitted ICU under tracheal intubation and mechanical ventilation. None DVT was detected.	ICU patients: 100%; BMI＞30: 31.25%
Tavazzi G, et al	Among 54 patients consecutively admitted to the ICU, 2 had PE and 8 had DVT.	ICU patients: 22.2%
Xing CY, et al	Vascular ultrasound was also performed to detect potential deep vein thrombosis. Among 20 patients, 7 patients developed DVT.	Not reported

Table S3. Pharmacologic thromboprophylaxis information of the included studies

Study	Population	Pharmacologic thromboprophylaxis information	Pharmacologic thromboprophylaxis rate	Definition
Beun R, et al	ICU	All VTE patients therapeutically treated with either unfractionated heparin intravenously (UFH) (aPTT ratio range between 2.0 and 3.0) or Low Molecular Weight Heparin (LMWH).	Not reported	NA
Bi QF, et al	mild-moderate and severe-critical	Not reported	Not reported	NA
Chen JP, et al	mild-moderate and severe-critical	Twenty patients were prophylactically treated with anticoagulant therapy (LMWH, 0.6mg/kg per 12 hours), and underwent a follow-up D-dimer test afterwards.	80% (20/25)	High
Cui SP, et al	ICU	No preventive anticoagulant was administered.	0%	Low
Ding Y, et al	NR	Not reported	Not reported	NA
Grillet F, et al	Non-ICU and ICU	Not reported	Not reported	NA
Helms J, et al	ICU	66.7% of patients were prophylactically treated with anticoagulant therapy (4000 UI/day for LMWH or if contra-indicated, UFH at 5–8 U/kg/h).	66.7%	High
Klok FA, et al	ICU	All patients received at least standard doses thromboprophylaxis (Leiden University Medical Center: nadroparin 2850 IU per day or 5700 IU per day if body	100%	High

		weight>100 kg; Erasmus University Medical Center: Nadroparin 5700 IU per day; nadroparin 5700 IU twice daily from April 4, 2020 and onwards; Amphia Hospital Breda: Nadroparin 2850 IU per day or 5700 IU per day if body weight >100kg; Nadroparin 5700 IU per day from March 30, 2020 and onwards). 17 patients (9.2%) received therapeutic anticoagulation at admission.		
Li T, et al	Suspected PE	Not reported	Not reported	NA
Llitjos JF, et al	ICU	31% (n=8) of patients received prophylactic anticoagulation. Patients treated with therapeutic anticoagulation received either LMWH or UFH with anti-Xa monitoring, with therapeutic levels of 0.3-0.7 U/mL of anti-Xa activity.	31%	High
Lodigiani C, et al	Non-ICU and ICU	All ICU patients received thromboprophylaxis with LMWH: the dosage was weight-adjusted in 17 patients and therapeutic in two patients on ambulatory treatment with direct oral anticoagulants. A total of 246 patients admitted to general wards received initial in-hospital thromboprophylaxis: a prophylactic dosage was used in 133 patients, 67 were treated with intermediate-dosage thromboprophylaxis, and 74 received therapeutic-dose	ICU: 100% (48/48);<br>General wards: 78.3% (246/314);<br>Overall: 81.2% (294/362)	High

		anticoagulation, including 22 who continued ambulatory treatment for atrial fibrillation or prior VTE.		
Lorant IL, et al	Non-ICU and ICU	42 patients received thromboembolic prophylaxis before CTPA. 7 patients received anticoagulation before CTPA.	39.6% (42/106)	Low
Middeldorp S, et al	Non-ICU and ICU	Thrombosis prophylaxis was part of standard of care in all COVID-19 patients. Ward patients received thrombosis prophylaxis with nadroparin 2,850 IU once-daily or 5,700 IU for patients with a body weight of ≥100 kg. From April 3 onwards, patients in ICU received a double dose of nadroparin as compared to patients on the wards, which was nadroparin 2,850 IU twice-daily for patients with a body weight <100 kg and 5,700 IU bid for those ≥100 kg. Thrombosis prophylaxis was initiated in 167 patients while 19 continued therapeutic anticoagulation for an indication that was present at the time of admission (e.g. atrial fibrillation). All VTE were diagnosed in patients receiving thrombosis prophylaxis. The risk of VTE in ICU patients was not lower during the period when the	100%	High

		standard dose of nadroparin prophylaxis was doubled than in the first follow-up period.		
Poissy J, et al	ICU	At the time of PE diagnosis, 20/22 patients were receiving prophylactic antithrombotic treatment (UFH or LWMH). One patient with a history of DVT was receiving fluindione with INR in the therapeutic range and one patient was receiving therapeutic UFH because of atrial fibrillation.	Not reported	NA
Ranucci M, et al	ICU	At the ICU admission, all the patients were receiving a thromboprophylaxis management of 4000 IU b.i.d. LWMH. After the first round of standard coagulation and viscoelastic tests, the patients were switched to the following protocol: LMWH 6000 b.i.d. (8000 IU b.i.d. if body mass index > 35).	100%	High
Tavazzi G, et al	ICU	All patients were sedated, mechanically ventilated and treated with prophylactic LMWH adjusted on body weight since the admission.	100%	High
Xing CY, et al	Moderate and severe-critical	Not reported	Not reported	NA

Table S4. Quality scores of the included studies

Study	Representativeness of the cases	Ascertainment of exposure	Ascertainment of outcome	Ascertainment of outcome (quality control) [a]	Control for factors of age and sex [b]	Control for factors related to VTE [c]	Total score
Beun R, et al(Beun et al., 2020)	1	1	1	1	2	1	7
Bi QF, et al(Bi et al., 2020)	1	1	1	1	1	2	7
Chen JP, et al(Chen et al., 2020)	1	1	1	1	2	2	8
Cui SP, et al(Cui et al., 2020)	1	1	1	1	2	2	8
Ding Y, et al(Ding et al., 2020)	1	1	1	1	2	0	6
Grillet F, et al(Grillet et al., 2020)	1	1	1	1	2	1	7
Helms J, et al(Helms et al., 2020)	1	1	1	1	2	1	7
Klok FA, et al(Klok et al., 2020)	1	1	1	1	2	1	7
Li T, et al(Li et al., 2020)	1	1	1	1	2	2	8

Llitjos JF, et al(Llitjos et al., 2020)	1	1	1	1	2	2	8
Lodigiani C, et al(Lodigiani et al., 2020)	1	1	1	1	2	2	8
Lorant IL, et al(Leonard-Lorant, 2020)	1	1	1	1	2	1	7
Middeldorp S, et al(Middeldorp, 2020)	1	1	1	1	2	2	8
Poissy J, et al(Poissy et al., 2020)	1	1	1	1	0	1	5
Ranucci M, et al(Ranucci et al., 2020)	1	1	1	1	2	1	7
Tavazzi G, et al(Kuo et al., 2020)	1	1	1	1	2	1	7
Xing CY, et al(Xing et al., 2020)	1	1	1	1	1	0	5

a, one point for studies that reported the diagnostic criteria of venous thromboembolism; b, one point for age, and one point for sex, totally 2 points for this section; c, studies received 1 point for reporting 1 or 2 categories, received 2 points for reporting ≥3 categories (previous VTE, hypertension, diabetes, cancer, anticoagulation rate, et al); VTE, venous thromboembolism

Table S5. Leave-1-out sensitivity analysis for VTE incidence

	Incidence (95% CI)		
**Study omitted**	**A. VTE**	**B. PE**	**C. DVT**
Beun R, et al	0.22 (0.16-0.28)	0.17 (0.11-0.22)	0.07 (0.04-0.10)
Bi QF, et al	0.28 (0.20-0.35)		0.10 (0.06-0.14)
Chen JP, et al	0.24 (0.18-0.30)	0.18 (0.12-0.24)	
Cui SP, et al	0.25 (0.19-0.31)		0.06 (0.03-0.08)
Ding Y, et al	0.27 (0.20-0.34)	0.21 (0.15-0.28)	
Grillet F, et al	0.25 (0.19-0.31)	0.19 (0.13-0.25)	
Helms J, et al	0.24 (0.18-0.31)	0.19 (0.13-0.24)	0.08 (0.05-0.11)
Klok FA, et al	0.26 (0.19-0.32)	0.20 (0.14-0.26)	0.09 (0.05-0.12)
Li T, et al	0.24 (0.18-0.30)	0.18 (0.12-0.24)	0.07 (0.04-0.10)
Llitjos JF, et al	0.22 (0.17-0.28)	0.19 (0.13-0.25)	
Lodigiani C, et al	0.28 (0.20-0.35)	0.22 (0.15-0.29)	0.09 (0.06-0.13)
Lorant IL, et al	0.24 (0.18-0.30)	0.18 (0.12-0.24)	
Middeldorp S, et al	0.25 (0.19-0.31)	0.21 (0.14-0.28)	0.06 (0.03-0.09)
Poissy J, et al	0.25 (0.19-0.31)	0.19 (0.13-0.25)	0.08 (0.05-0.11)
Ranucci M, et al	0.25 (0.19-0.31)	0.19 (0.13-0.25)	0.07 (0.04-0.10)
Tavazzi G, et al	0.25 (0.19-0.32)	0.21 (0.15-0.28)	0.07 (0.04-0.10)
Xing CY, et al	0.24 (0.18-0.31)		0.07 (0.04-0.10)

CI, confidence interval; VTE, venous thromboembolism; PE, pulmonary embolism; DVT, deep venous thrombosis

Table S6. Leave-1-out sensitivity analysis for relative risk of severe patients vs. non-severe patients

**Study omitted**	**RR (95% CI)**
Bi QF, et al(Bi et al., 2020)	4.36 (2.56-7.44)
Chen JP, et al(Chen et al., 2020)	5.26 (2.64-10.46)
Grillet F, et al(Grillet et al., 2020)	4.96 (2.36-10.50)
Lodigiani C, et al(Lodigiani et al., 2020)	5.47 (2.93-10.19)
Lorant IL, et al(Leonard-Lorant, 2020)	5.26 (2.47-11.22)
Middeldorp S, et al(Middeldorp, 2020)	3.66 (2.40-5.57)
Xing CY, et al(Xing et al., 2020)	4.82 (2.57-9.03)

RR, relative risk; CI, confidence interval

Table S7. Univariable meta-regression for VTE incidence

**Population**	**Variables**	**No. of reported studies**	**β coefficient (95%CI)**	***P* value**
VTE	Mean age	12	0.0045(-0.0375-0.0464)	0.817
	Male	14	-0.0001 (-0.0099-0.0097)	0.986
	VTE	7	-0.0126 (-0.0960-0.0708)	0.714
	Hypertension	6	-0.0023 (-0.0121-0.0036)	0.559
	Diabetes	5	-0.0026 (-0.0673-0.0621)	0.906
	Cancer	5	-0.0001 (-0.0415-0.0415)	0.998
	Anticoagulation	9	-0.0002 (-0.0046-0.0043)	0.928
PE	Mean age	10	0.0164 (-0.0637-0.0964)	0.650
	Male	10	0.0014 (-0.0139-0.0167)	0.837
	VTE	7	-0.0008 (-0.1082-0.1066)	0.985
	Hypertension	4	0.0017 (-0.0425-0.0459)	0.885
	Diabetes	3	0.0539 (-1.8031-1.9110)	0.775
	Cancer	4	-0.0026 (-0.0654-0.0601)	0.873
	Anticoagulation	7	0.0009 (-0.0080-0.0097)	0.813
DVT	Mean age	5	0.0116 (-0.0877-0.1110)	0.734
	Male	7	-0.0008 (-0.0204-0.0221)	0.922
	VTE	4	-0.0697 (-0.4408-0.3014)	0.504
	Hypertension	4	-0.0007 (-0.0212-0.0199)	0.900

	Diabetes	3	0.0133 (-0.5209-0.5475)	0.805
	Cancer	3	-0.0060 (-1.6036-1.5915)	0.970
	Anticoagulation	5	0.0004 (-0.0077-0.0085)	0.881

No, number; CI, confidence interval; VTE, venous thromboembolism; PE, pulmonary embolism; DVT, deep venous thrombosis

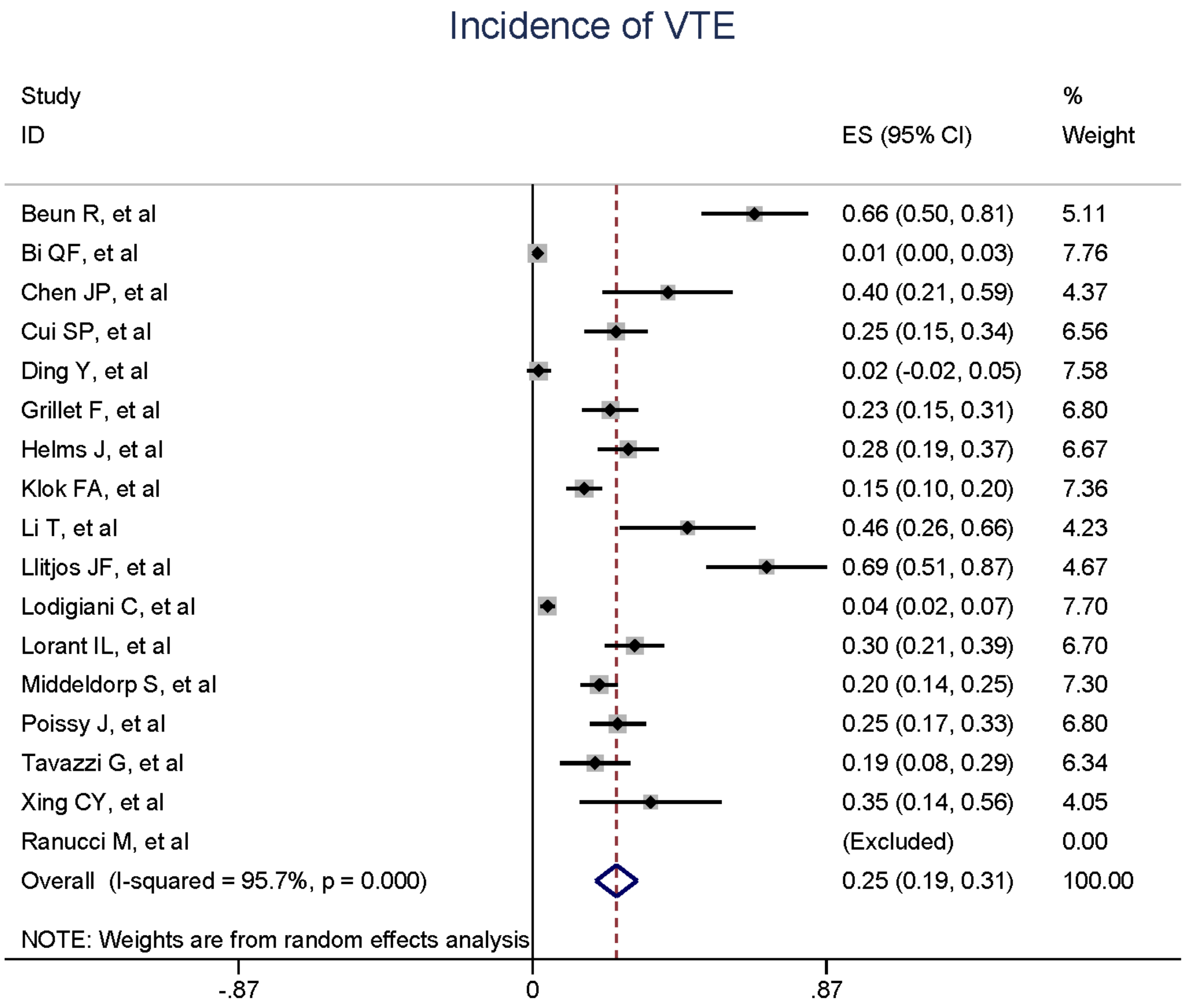

Figure S1. Incidence of venous thromboembolism

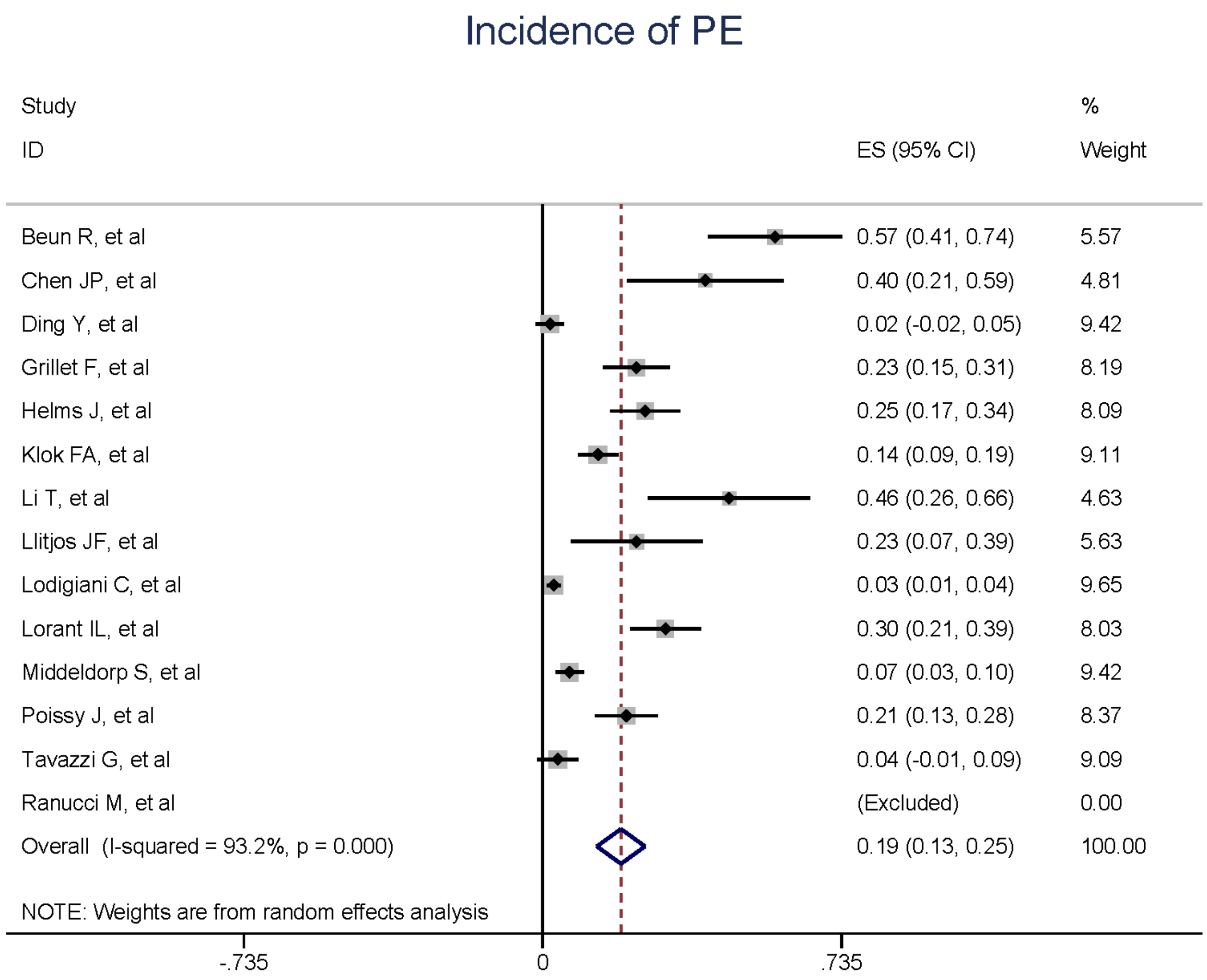

Figure S2. Incidence of pulmonary embolism

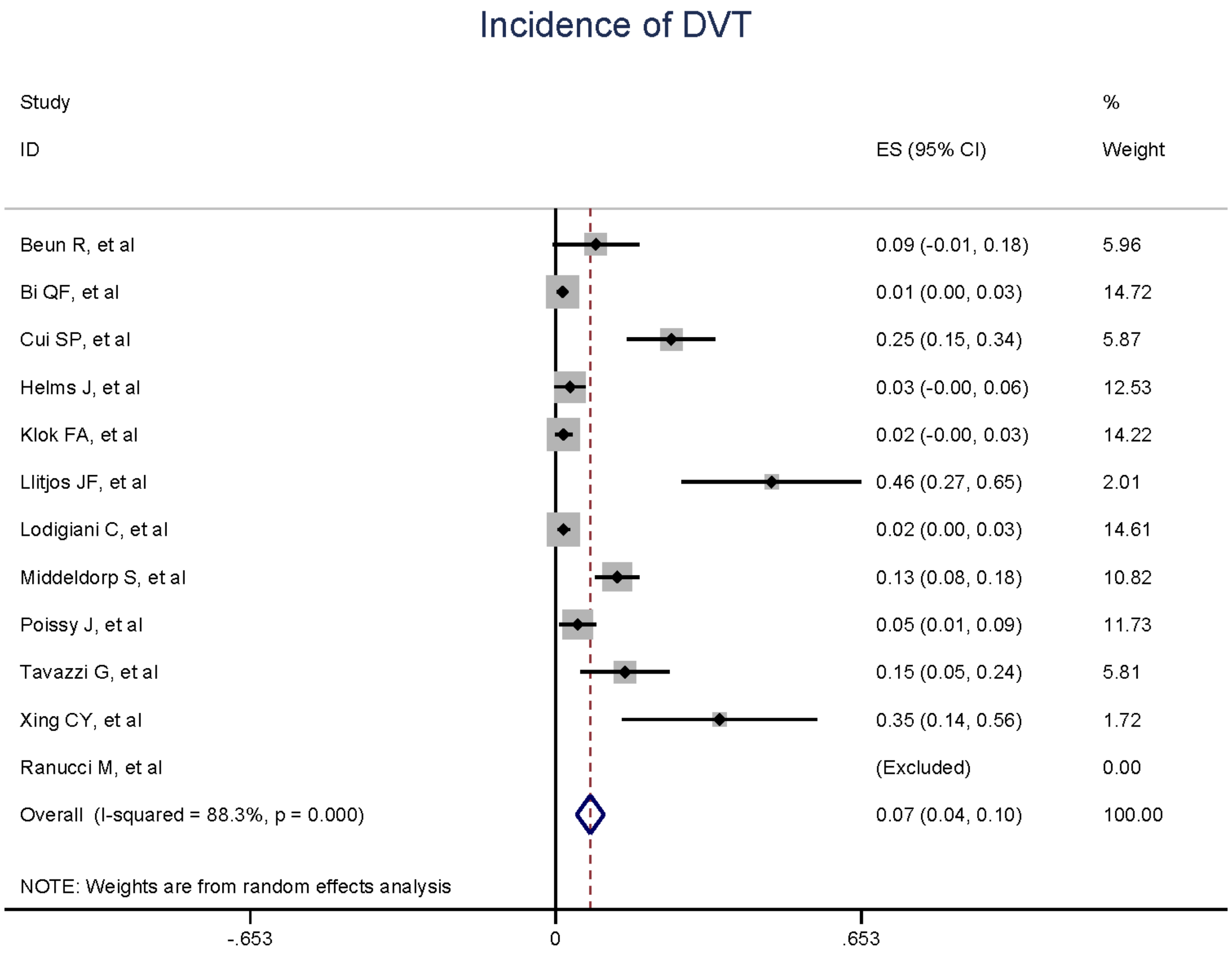

Figure S3. Incidence of deep vein thrombosis

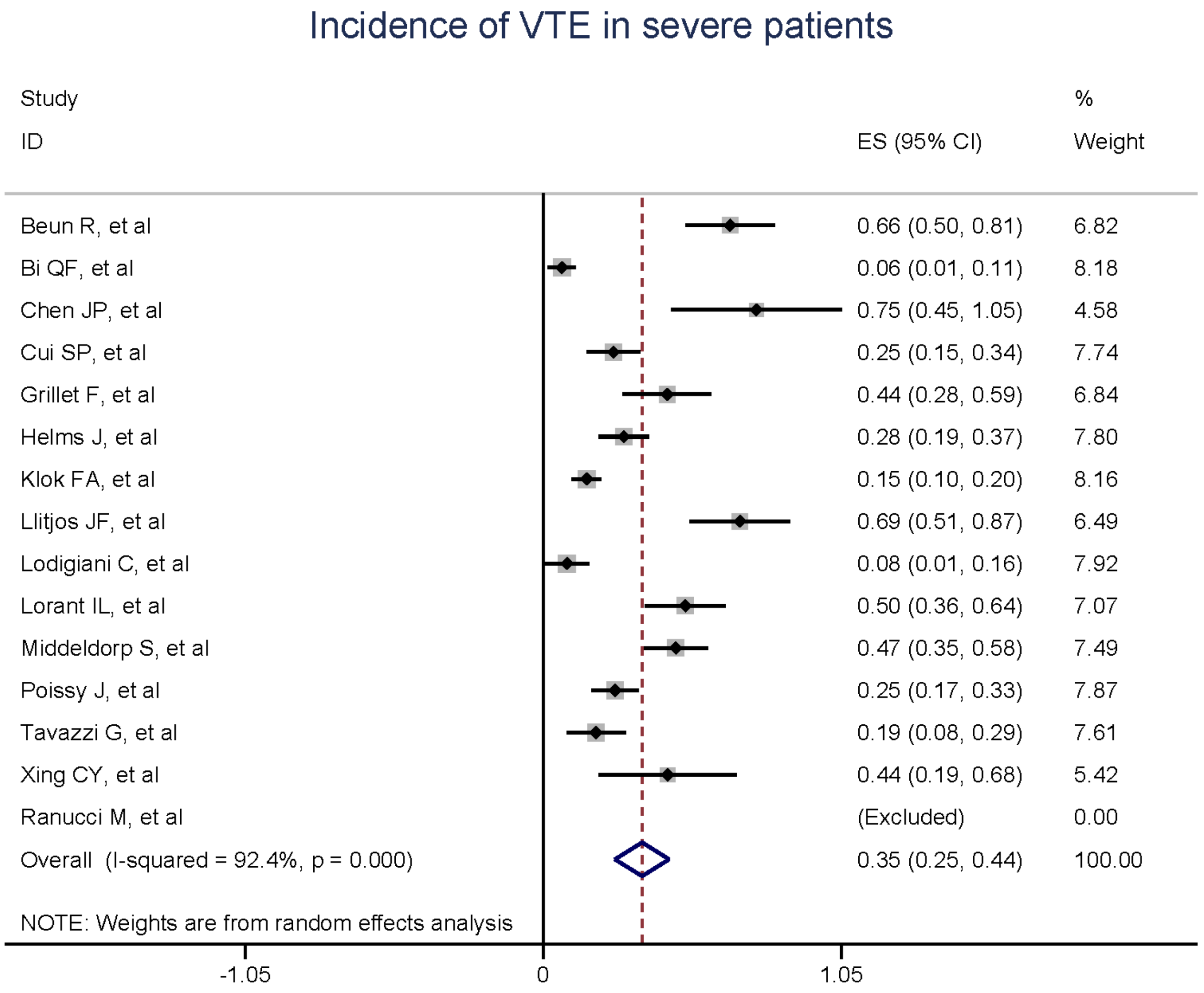

Figure S4. Incidence of venous thromboembolism in severe patients

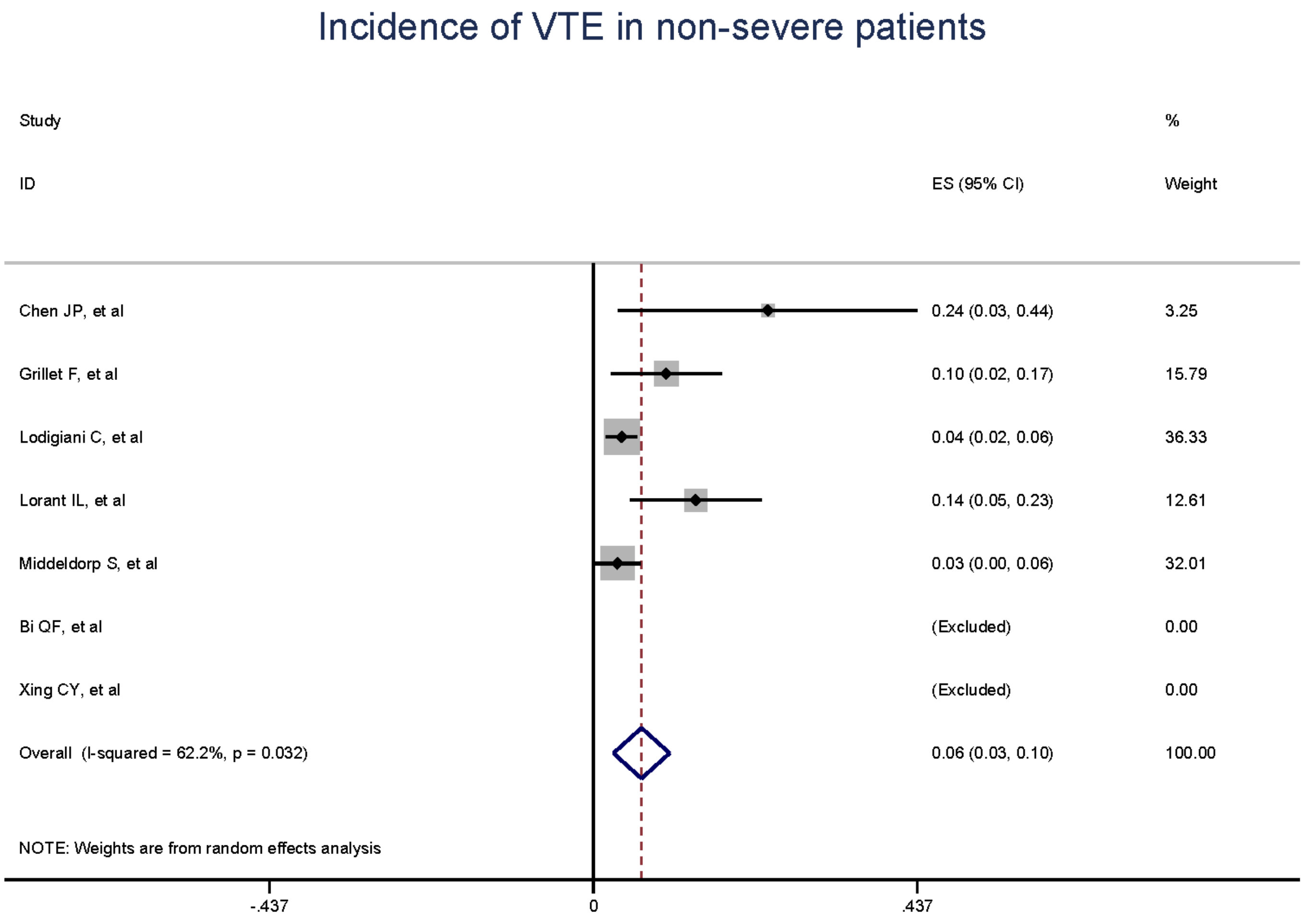

Figure S5. Incidence of venous thromboembolism in non-severe patients

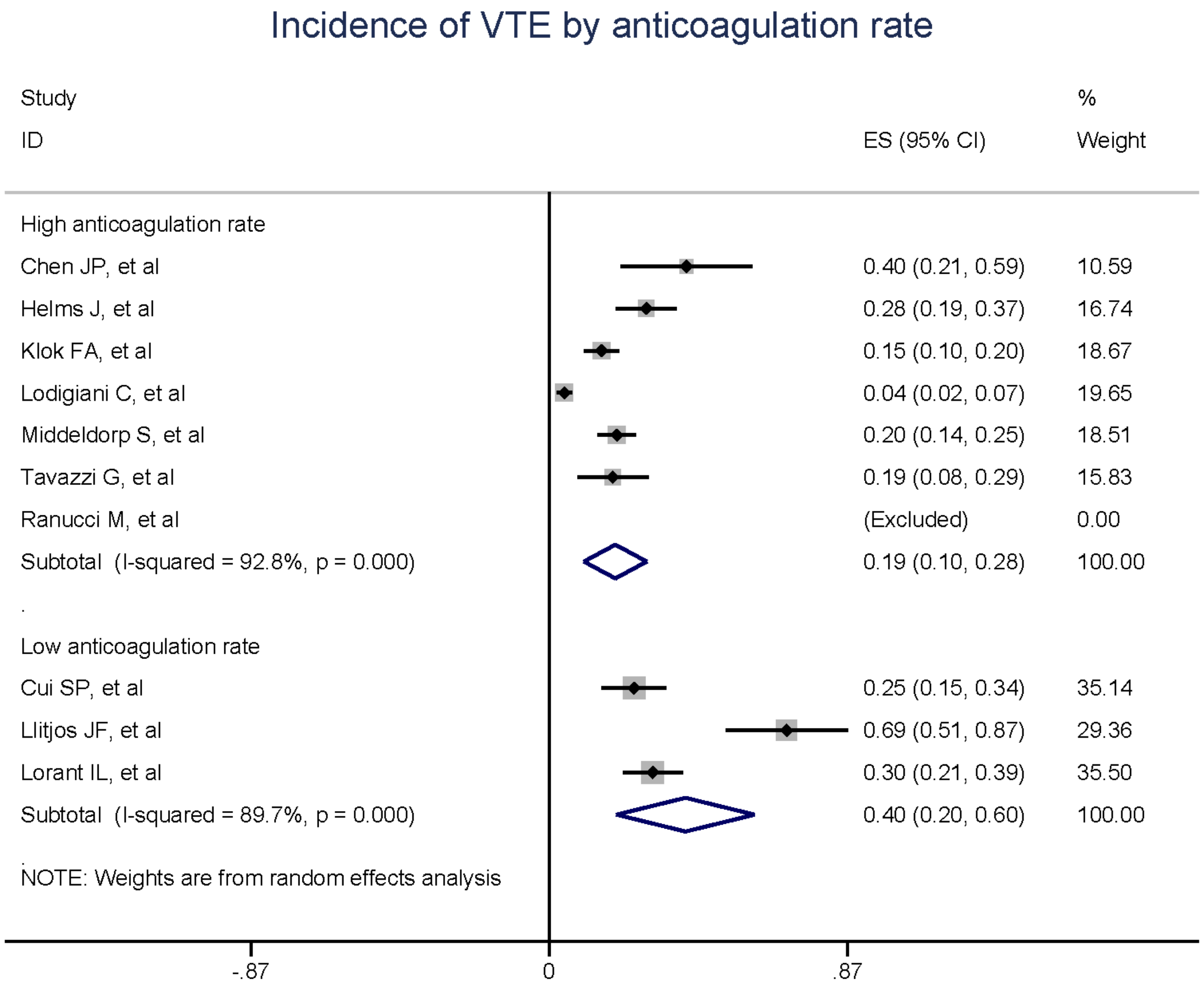

Figure S6. Incidence of venous thromboembolism by anticoagulation rate

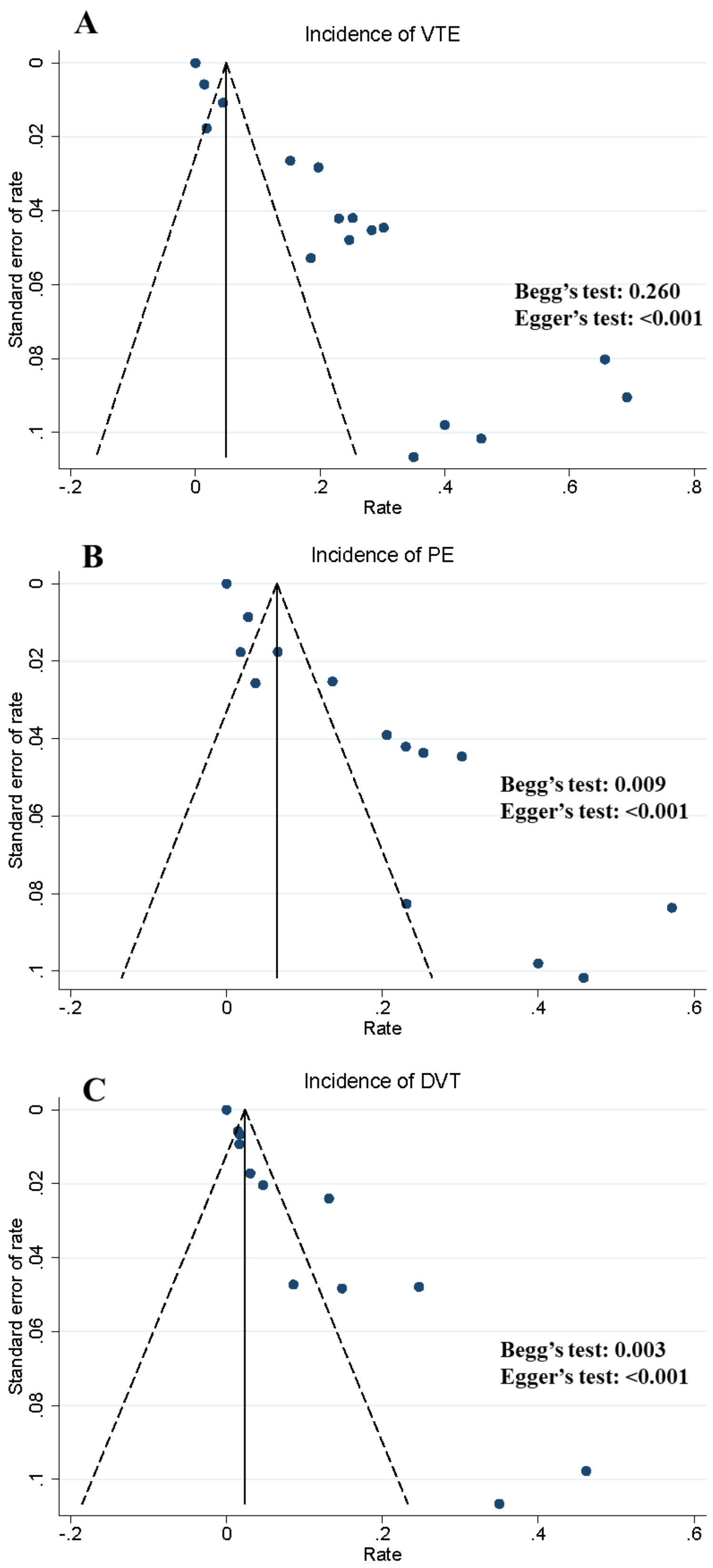

Figure S7. Publication bias on the incidence of venous thromboembolism (A. venous thromboembolism; B. pulmonary embolism; C. deep venous thrombosis)

References:

Beun, R., Kusadasi, N., Sikma, M., Westerink, J., and Huisman, A. (2020). Thromboembolic events and apparent heparin resistance in patients infected with SARS-CoV-2. doi: 10.1111/ijlh.13230.

Bi, Q., Hong, C., Meng, J., Wu, Z., Zhou, P., Ye, C., et al. (2020). Characterization of clinical progression of COVID-19 patients in Shenzhen, China. 2020.2004.2022.20076190. doi: 10.1101/2020.04.22.20076190 %J medRxiv.

Chen, J., Wang, X., Zhang, S., Liu, B., Wu, X., Wang, Y., et al. (2020). Findings of acute pulmonary embolism in COVID-19 patients. *The Lancet Infectious Diseases*.

Cui, S., Chen, S., Li, X., Liu, S., and Wang, F. (2020). Prevalence of venous thromboembolism in patients with severe novel coronavirus pneumonia. *J Thromb Haemost*. doi: 10.1111/jth.14830.

Ding, Y., Huang, Z., Zhao, S., Li, X., Wang, X., and Xie, Y. (2020). Clinical and imaging characteristics of corona virus disease 2019(COVID-19). *Radiol Practice* 35(3)**,** 281-285. doi: 10.13609/j.cnki.1000-0313.2020.03.008.

Grillet, F., Behr, J., Calame, P., and Aubry, S. (2020). Acute Pulmonary Embolism Associated with COVID-19 Pneumonia Detected by Pulmonary CT Angiography. *Radiology***,** 201544. doi: 10.1148/radiol.2020201544.

Helms, J., Tacquard, C., Severac, F., Leonard-Lorant, I., Ohana, M., Delabranche, X., et al. (2020). High risk of thrombosis in patients with severe SARS-CoV-2 infection: a multicenter prospective cohort study. *J Thromb Thrombolysis*. doi: 10.1007/s11239-020-02125-4

10.1007/s00134-020-06062-x.

Klok, F.A., Kruip, M., van der Meer, N.J.M., Arbous, M.S., Gommers, D., Kant, K.M., et al. (2020). Incidence of thrombotic complications in critically ill ICU patients with COVID-19. *Thromb Res*. doi: 10.1016/j.thromres.2020.04.013.

Kuo, J.S., Shaikh, H.A., Tavazzi, G., Civardi, L., Caneva, L., Mongodi, S., et al. (2020). Thrombotic events in SARS-CoV-2 patients: an urgent call for ultrasound screening. *Radiology*. doi: 10.1148/radiol.2020201623

10.1007/s00134-020-06040-3.

Leonard-Lorant, I. (2020). Acute Pulmonary Embolism in COVID-19 Patients on CT Angiography and Relationship to D-Dimer Levels. *Radiology***,** 201561. doi: 10.1148/radiol.2020201544

10.1148/radiol.2020201561.

Li, T., Kicska, G., Kinahan, P.E., Zhu, C., Oztek, M.A., and Wu, W. (2020). Clinical and Imaging Findings in COVID-19 Patients Complicated by Pulmonary Embolism. 2020.2004.2020.20064105. doi: 10.1101/2020.04.20.20064105 %J medRxiv.

Llitjos, J.F., Leclerc, M., Chochois, C., Monsallier, J.M., Ramakers, M., Auvray, M., et al. (2020). High incidence of venous thromboembolic events in anticoagulated severe COVID-19 patients. *J Thromb Haemost*. doi: 10.1111/jth.14869.

Lodigiani, C., Iapichino, G., Carenzo, L., Cecconi, M., Ferrazzi, P., Sebastian, T., et al. (2020). Venous and arterial thromboembolic complications in COVID-19 patients admitted to an academic hospital in Milan, Italy. *Thromb Res* 191**,** 9-14. doi: 10.1016/j.thromres.2020.04.024.

Middeldorp, S. (2020). Incidence of venous thromboembolism in hospitalized patients with COVID-19. *J Investig Med High Impact Case Rep*. doi: 10.1177/2324709620925571

10.1111/jth.14888.

Poissy, J., Goutay, J., Caplan, M., Parmentier, E., Duburcq, T., Lassalle, F., et al. (2020). Pulmonary Embolism in COVID-19 Patients: Awareness of an Increased Prevalence. *Circulation*. doi: 10.1093/jtm/taaa063

10.1161/circulationaha.120.047430.

Ranucci, M., Ballotta, A., Di Dedda, U., Bayshnikova, E., Dei Poli, M., Resta, M., et al. (2020). The procoagulant pattern of patients with COVID-19 acute respiratory distress syndrome. *J Thromb Haemost*. doi: 10.1111/jth.14854.

Xing, C., Li, Q., Du, H., Kang, W., Lian, J., and Yuan, L. (2020). Lung ultrasound findings in patients with COVID-19 pneumonia. *Crit Care* 24(1)**,** 174. doi: 10.1186/s13054-020-02876-9.
